# Antioxidant Activity of Natural Hydroquinones

**DOI:** 10.3390/antiox11020343

**Published:** 2022-02-09

**Authors:** Rosa M. Giner, José Luis Ríos, Salvador Máñez

**Affiliations:** Departament de Farmacologia, Facultat de Farmàcia, Universitat de València, Av. Vicent Andrés Estellés s/n, 46100 Burjassot, Spain; rmginer@uv.es (R.M.G.); riosjl@uv.es (J.L.R.)

**Keywords:** alkyl phenolics, antioxidant, hydroquinones, marine natural products, meroterpenoids, reactive oxygen species, secondary metabolites

## Abstract

Secondary metabolites derived from hydroquinone are quite rare in nature despite the original simplicity of its structure, especially when compared to other derivatives with which it shares biosynthetic pathways. However, its presence in a prenylated form is somewhat relevant, especially in the marine environment, where it is found in different algae and invertebrates. Sometimes, more complex molecules have also been identified, as in the case of polycyclic diterpenes, such as those possessing an abietane skeleton. In every case, the presence of the dihydroxy group in the *para* position gives them antioxidant capacity, through its transformation into *para*-quinones.This review focuses on natural hydroquinones with antioxidant properties referenced in the last fifteen years. This activity, which has been generally demonstrated in vitro, should lead to relevant pharmacological properties, through its interaction with enzymes, transcription factors and other proteins, which may be particularly relevant for the prevention of degenerative diseases of the central nervous system, or also in cancer and metabolic or immune diseases. As a conclusion, this review has updated the pharmacological potential of hydroquinone derivatives, despite the fact that only a small number of molecules are known as active principles in established medicinal plants. The highlights of the present review are as follows: (a) sesquiterpenoid zonarol and analogs, whose activity is based on the stimulation of the Nrf2/ARE pathway, have a neuroprotective effect; (b) the research on pestalotioquinol and analogs (aromatic ene-ynes) in the pharmacology of atherosclerosis is of great value, due to their agonistic interaction with LXRα; and (c) prenylhydroquinones with a selective effect on tyrosine nitration or protein carbonylation may be of interest in the control of post-translational protein modifications, which usually appear in chronic inflammatory diseases.

## 1. Introduction

**Figure 1:**
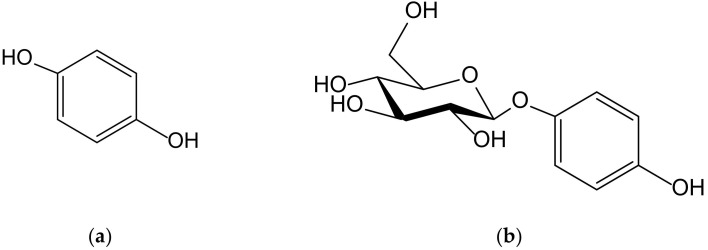
Chemical structures of hydroquinone (**a**) and arbutin (**b**).

Hydroquinone (1,4-dihydroxybenzene, Figure 1) is a very simple phenolic structure that appears in secondary plant metabolites present in species with different phylogenies, with the most prominent taxa being found in Ericales, Lamiales and Asterales. However, such compounds are present in brown and green algae and in bacteria and a high number of marine invertebrate organisms, such as sponges, cnidaria, and ascidians.

Although an exhaustive study of the biosynthetic origin of hydroquinones is outside the scope of this review, it is perhaps pertinent to mention a few general lines. At first, it must be stated that the *p*-dihydroxybenzene ring comes from the shikimate pathway, from chorismate to *p*-hydroxybenzoate (PHB), by the effect of chorismate lyase, then by the subsequent transformation into hydroquinone by PHB-1-hydroxylase, as it has been analyzed in detail in the process of heterologous production of arbutin, also termed arbutoside (4-hydroxyphenyl-1-*O*-β-D-glucopyranoside, Figure 1), in *Yarrowia* [1]. The transformation of homogentisate into gentisate and the decarboxylation of gentisate into hydroquinone constitute alternative, less frequent routes, as occurs in sponge prenyl-hydroquinones [2]. Secondly, it should be noted that many of the natural hydroquinones have a terpenoid part, which is sometimes very bulky, which is why they are considered part of the complex group of meroterpenoids. As regards the way in which the aromatic ring is integrated into these types of molecules, in most cases it is a simple conjunction driven by prenyl transferases, but on other occasions such a ring is a part of its own terpenoid skeleton and, therefore, the benzene ring is generated by cyclic dehydrogenations.

Hydroquinone derivatives, when both hydroxyls are free, are easily transformed, in the presence of oxygen or metal cations, into *p*-quinones. The transit between the phenolic (reduced) and quinonic (oxidized) forms involves the participation of 2H^+^ and 2e^−^, and the redox potential of the process is markedly influenced by the pH of the medium and the nature of the solvent. For the purposes of biological activity, it is important to note that the aforementioned reaction is carried out in two steps, with the formation of an intermediate semiquinone, a compound endowed with a certain capacity to generate reactive oxygen species (ROS), which is very relevant to possible cellular toxicity [3]. For decades, this concept has been crucial in the field of mitochondrial biochemistry since the functioning of the complexes I and III of the respiratory chain is based on this reaction, materialized by the pair ubiquinone/ubiquinol (coenzyme Q), as was described by Frederick Crane [4].

On the basis of this set of concepts, the present review tries to systematize the studies on the antioxidant effects of natural hydroquinones by analyzing the most important papers published during the 2005–2020 period, with some temporal extension, when applicable. The organization of this manuscript is based on two criteria: the analytical methodology concerning the oxidative processes and the chemical structure of the active principles.

## 2. Free Radical Scavenging and Related Antioxidant Chemical Analysis

### 2.1. Simple Hydroquinones

In a study on the oxygen free radical scavenging properties of 70 Chinese medicinal plants, *Rhodiola sacra* (Prain ex Raym.-Hamet) S.H. Fu (Crassulaceae) was one of the most active, along with other species belonging to the well-known genera *Areca*, *Juglans*, *Paeonia* and *Psychotria*. Further research on the principles of the roots of *R. sacra* determined that hydroquinone exerted a quite moderate activity as a scavenger of the superoxide anion (O_2_^•^^−^) generated by a hypoxanthine/xanthine oxidase system and measured by spin trapping with 5,5-dimethylpyrroline-*N*-oxide (IC_50_ 72 µM). Moreover, some other common catechins and phenolic acids, as well as, to a lesser extent, the cyanogenic glycoside heterodendrin, participate in this effect, which was primarily observed in both methanolic and aqueous extracts of the crude drug. Provided that their IC_50_ values were ranged 1.5–4.0 µM, the principles responsible for the antioxidant effect of the *R. sacra* root extracts seem to be gallic acid and its catechin esters found therein [5]. 

The aerial parts of *Origanum majorana* L. (Lamiaceae) were extracted with boiling water for 2 h, and the aqueous extract fractionated with hexane and ethyl acetate. From the ethyl acetate extract, the following phenolic compounds were purified and identified: hydroquinone, arbutin, rosmarinic acid, 5,6,3′-trihydroxy-7,8,4′-trimethoxyflavone, and 5,7,3′-trihydroxy-4′-methoxyflavanone (hesperetin). The antioxidant activity was determined by carrying out two tests of free radical scavenging: 1,1-diphenyl-2-picrylhydrazylradical (DPPH^•^) assay and 2,2′-azinobis-(3-ethylbenzothiazoline-6-sulfonic acid) radical cation (ABTS^•+^), and the ferric reducing antioxidant power (FRAP) assay, with potassium ferricyanide. Among these compounds, hydroquinone (0.054% of plant dry weight) was the most potent in both tests, whereas its glucoside, arbutin (0.039% of plant dry weight), was the least potent. However, both hydroquinonic principles differed compared to the rest, for their highest anti-proliferative potency on tumoral C-6 cells. In order to get an appropriate picture of the relative influence of the different phenolic principles on the effects of *O. majorana* as a whole, some comparisons may be quite useful: the IC_50_ of hydroquinone in the DPPH test was 17.44 µM, whereas that of hesperetin (3.81% of plant dry weight) was 21.44 µM. Considering these data, the contribution of this last flavonoid to the plant activity, as a free radical scavenger agent, should be circa 20 times that of hydroquinone [6].

**Figure 2:**
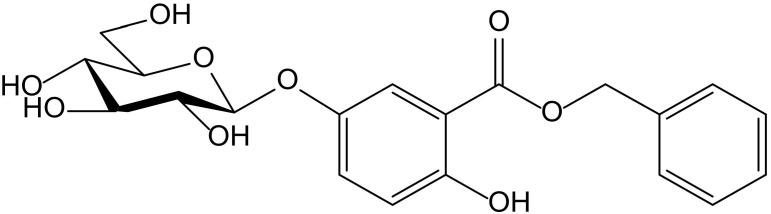
Chemical structure of benzyl 5-*O*-β-D-glucopyranosyl-2,5-dihydroxybenzoate isolated from *Mikania micrantha*.

From the aerial parts of *Mikania micrantha* Kunth (Compositae), Dong et al. identified fourteen phenolics, among them hydroquinone itself and the new hydroquinone derivative, benzyl 5-*O*-β-D-glucopyranosyl-2,5-dihydroxybenzoate (Figure 2). Both compounds were tested as potential antioxidant principles in ABTS^•+^ and DPPH^•^ radical scavenging assays, and in the FRAP assay, with L-ascorbic acid as a reference compound. In the case of the glucoside, when tested on the ABTS assay, it showed a scavenger concentration 50% (SC_50_) of 0.31 µM, the highest potency among the tested compounds, including hydroquinone (SC_50_ 4.57 µM) and ascorbic acid (SC_50_ 10.45 µM). In the FRAP assay, it also displayed interesting activity with a reducing capacity of 0.34 mmol/g, clearly better than the ones obtained for hydroquinone (8.77 mmol/g) and ascorbic acid (11.32 mmol/g). However, the glycoside had no effect in the DPPH test (SC_50_ > 100 µM), whereas hydroquinone (SC_50_ 31.96 µM) showed a potency similar to that of ascorbic acid (SC_50_ 39.48 µM). The authors proposed this compound for future studies on chronic diseases in which free radicals and ROS are implicated, such as atherosclerosis, angiocardiopathy, and cancer [7].

In a detailed work on the phenolic compounds of both raw and bee-processed corn pollen obtained from hives in the province of Nan (Thailand), hydroquinone and a glycoside of the flavone apigenin were described as the most powerful antioxidants therein. Nevertheless, the nuclear magnetic resonance spectroscopic data that might justify the identification of hydroquinone are inconclusive and, therefore, the actual presence of this molecule in the analyzed extracts seems to be untrue [8].

**Figure 3:**
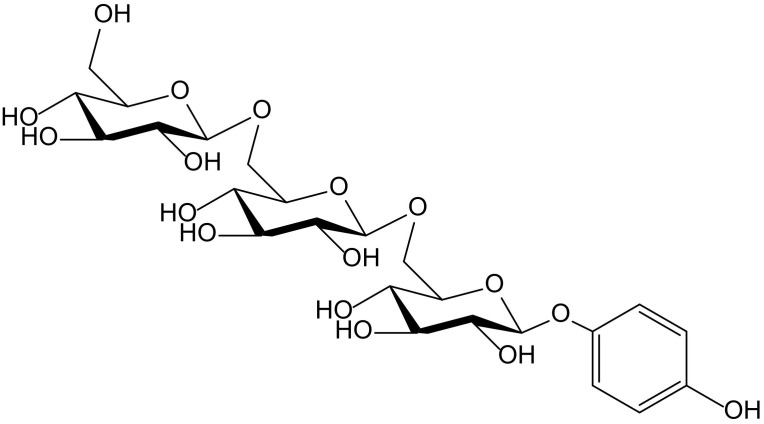
Chemical structure of 4-hydroxyphenyl-β-D-glucopyranosyl-(1→6)-β-D-glucopyranosyl-(1→6)-β-D-glucopyranoside isolated from *Triticum aestivum*.

The hydroquinone trisaccharide, 4-hydroxyphenyl-β-D-glucopyranosyl-(1→6)-β-D-glucopyranosyl-(1→6)-β-D-glucopyranoside (Figure 3), together with some other closely related hydroxybenzene glycosides, was isolated as a minor from the germs of wheat (*Triticum aestivum* L., Poaceae). That compound showed activity in the so called Trolox equivalent antioxidant capacity (TEAC) test, which consists of the oxidation of ABTS to form the free radical ABTS^•+^. The capture of ABTS^•+^ was monitored, in comparison to that produced by 6-hydroxy-2,5,7,8-tetramethylchroman-2-carboxylic acid (Trolox). Under such conditions, the hydroquinone triglucoside showed a softly growing effect along 5 min, with potency 1.12 times higher than that of Trolox. The authors managed to explain how, in previous papers, it was reported that an increase of the number of sugars in simple hydroquinones weakened the antioxidant activity in the DPPH scavenging test. This fact, which certainly does not occur with ABTS^•+^, probably correlates with the steric hindrance affecting DPPH^•^ radical [9].

**Figure 4:**
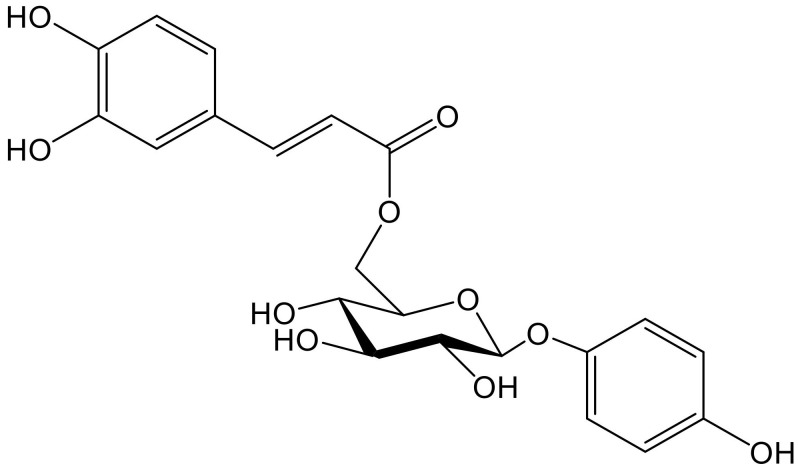
Chemical structure of robustaside B isolated from *Cnestis ferruginea*.

Hydroquinone and its 6″-*O*-caffeoylglucoside (robustaside B, Figure 4) were described in the leaves of *Cnestis ferruginea* Vahl ex DC. (Connaraceae), a medicinal plant native to western tropical Africa, known for its laxative and antimicrobial activity. Both phenolic principles were characterized as antioxidants when rat mitochondria were treated with Fe^2+^/ascorbate to induce membrane lipid peroxidation. At the concentrations tested (0.05–1.0 mM), there was no concentration–response relationship since the percentages of inhibition of the production of thiobarbituric acid reacting species (TBARS) only varied from 81.0 to 86.5. It is clear that some experiments working in the µM, even nM, range would have been advisable. Furthermore, hydroquinone and robustaside B caused the rat liver mitochondria permeability transition pore to open, although they acted in the opposite way when such opening was caused by an increase in calcium. In agreement with the latter effect, it should be noted that release of calcium provokes deregulation of membrane function, mitochondrial swelling, and cell apoptosis [10].

### 2.2. Terpenoid Hydroquinones

**Figure 5:**
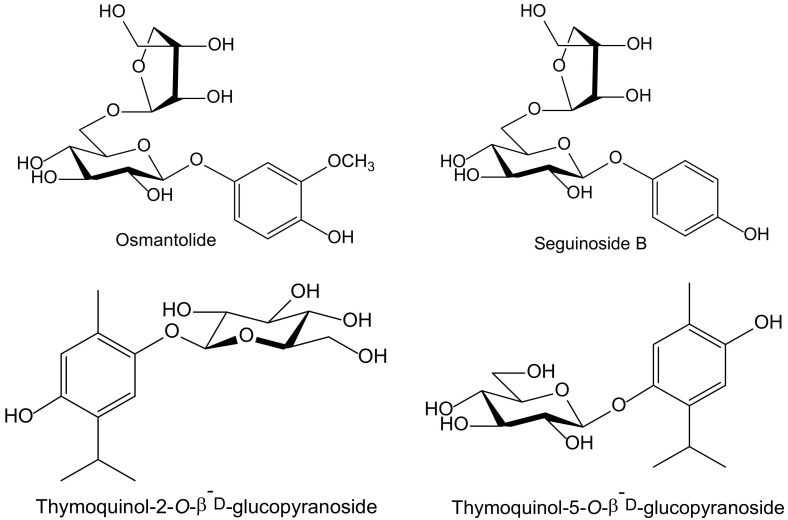
Chemical structures of hydroquinones isolated from *Origanum majorana*.

Of the eighteen compounds isolated from the aerial parts of *Origanum dubium* Boiss—in fact, a synonym of *O. majorana* (Lamiaceae)—collected in Cyprus, three were identified as simple hydroquinone glycosides: arbutin, seguinoside B, and osmantolide, and three more as monoterpenoid hydroquinone glycosides: thymoquinol-2-*O*-β-D-glucopyranoside, and thymoquinol-5-*O*-β-D-glucopyranoside (Figure 5). Osmantolide seems to be the principal active compound in the DPPH test since it showed, at 0.1 mM, a 63% and 65% inhibition at 20 and 60 min, respectively, whereas the other compounds assayed did notexceed 30% at similar concentrations. For the inhibition of linoleic acid peroxidation, the authors used 2,2′-azobis(2-amidinopropane) (ABAP) as a free radical initiator, and Trolox was used as a standard. Thymoquinol-5-*O*-β-D-glucopyranoside (85%) and osmantolide (77%) gave the highest inhibitory effects, whereas the rest of the compounds did notexceed 40%. The authors included some experimental data related to potential anti-inflammatory activity, but they did not perform any tests of inflammation [11].

**Figure 6:**
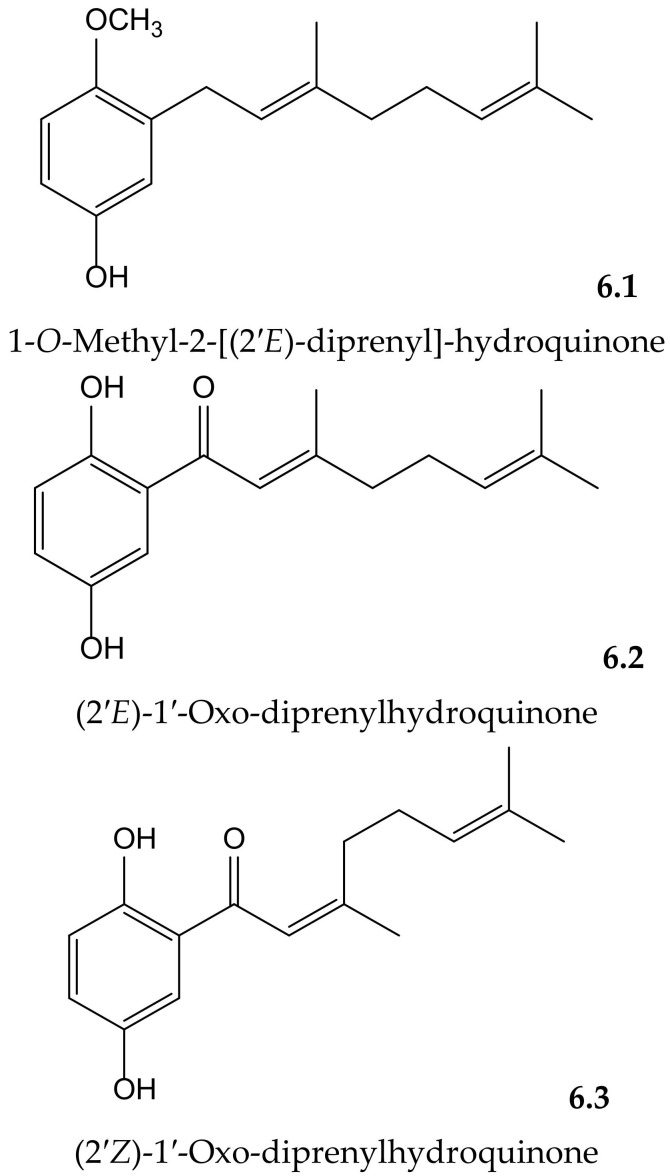
Chemical structures of hydroquinones isolated from *Piper crassinervium*.

Yamaguchi et al. isolated and identified the new 1-*O*-methyl-2-[(2′*E*)-diprenyl]-hydroquinone together with the known (2′E)-1′-oxo-diprenylhydroquinone and (2′*Z*)-1′-oxo-diprenylhydroquinone (Figure 6) from the fruits of *Piper crassinervium* Kunth (Piperaceae). They evaluated the antioxidant potential of these geranyl-hydroquinone derivatives through their capacity to inhibit both DPPH^•^ radical scavenging and luminol chemiluminescence, induced by ABAP as a peroxyl radical source. Their protective effects against lipid peroxidation induced by Fe^3+^/EDTA and ascorbic acid were assessed in liposomes of phosphatidylcholine. These prenylated hydroquinones exhibited substantial radical scavenger capacities in the DPPH^•^, chemiluminescence, and lipid peroxidation tests. The new natural compound showed higher potency (IC_50_ 14.5 μM) for lipid peroxidation than the known ones (**6.2** and **6.3**, with IC_50_ 26.4 and 63.1 μM, respectively) but lower potency than Trolox (IC_50_ 2.6 μM). Among these last hydroquinones, the *E* isomer was more efficient than *Z* isomer, probably due to its better insertion in the liposome [12].

**Figure 7:**
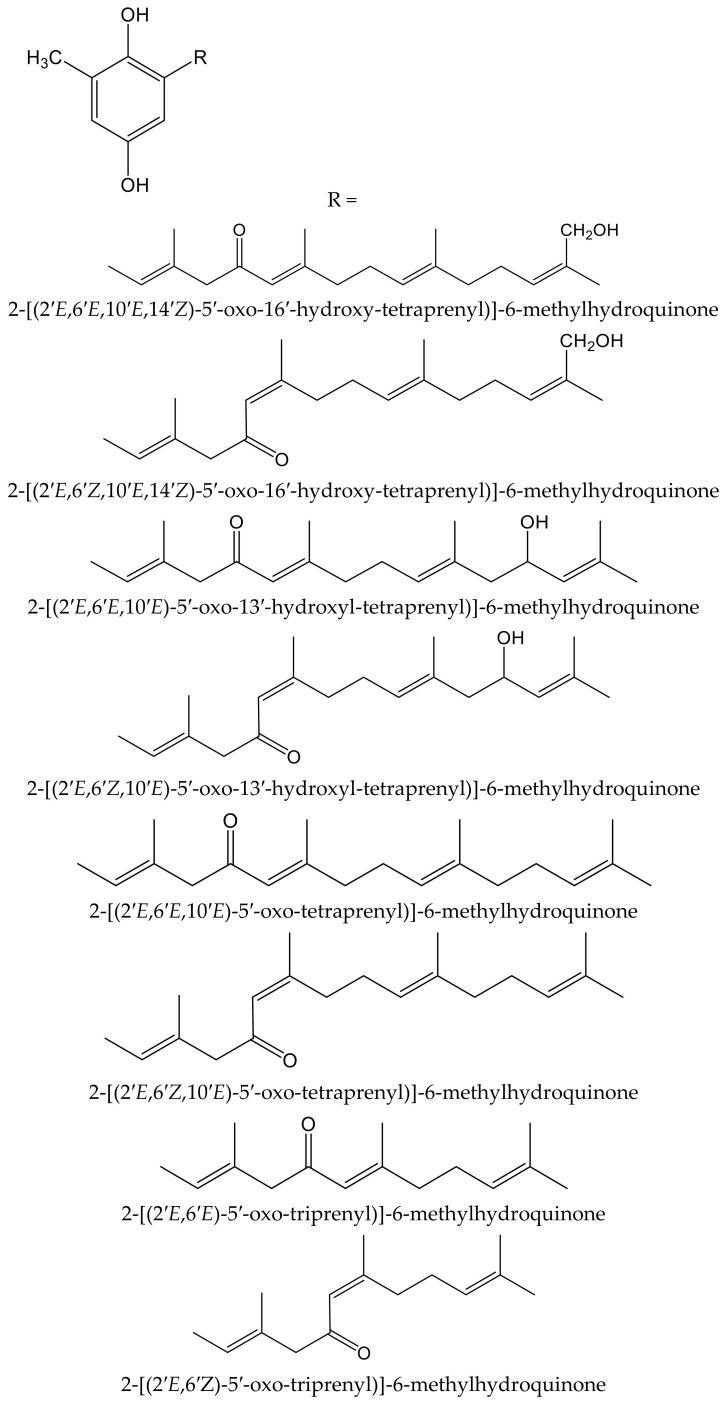
Chemical structures of the new hydroquinones isolated from *Cystoseiracrinita*.

Algae are another source of hydroquinones and related compounds. Fish et al. isolated fourteen meroterpenoids from the brown alga *Cystoseira crinita* Duby (Sargassaceae). Eight of them were new prenyl-toluquinols, a class of methylhydroquinone compounds, with six of them being tetraprenyl derivatives and two triprenyl derivatives (Figure 7). Furthermore, some additional known, related molecules were identified. All these compounds were tested for their antioxidant properties in the DPPH and TBARS tests. In the DPPH test, the hydroquinone derivatives gave the highest radical-scavenging effects, with percentages of scavenging activities between 93 and 97%, similar to those of α-tocopherol (95%), all of them at 230 µM. At lower concentrations, only three compounds had remarkable activity, with scavenging activities of 80% at 58 µM and 30% at 23 µM. In the TBARS assay of peroxidation of linolenic acid methyl ester, all hydroquinones had similar effect to that of the control, being 69% for butylated hydroxytoluene (BHT) and 73% for α-tocopherol. In the TEAC test, they showed activities between 13% and 59% that of α-tocopherol, whereas in the photochemiluminescence test the highest values were 41% and 112% that of α-tocopherol, which indicated that some of these compounds had a relevant radical-scavenging power. Despite the high number and variety of the analyzed compounds, it has not been possible to establish either the influence of the radicals nor other structural activity relationships; so, it was considered that the only chemical group of interest as an antioxidant is hydroquinone [13]. 

**Figure 8:**
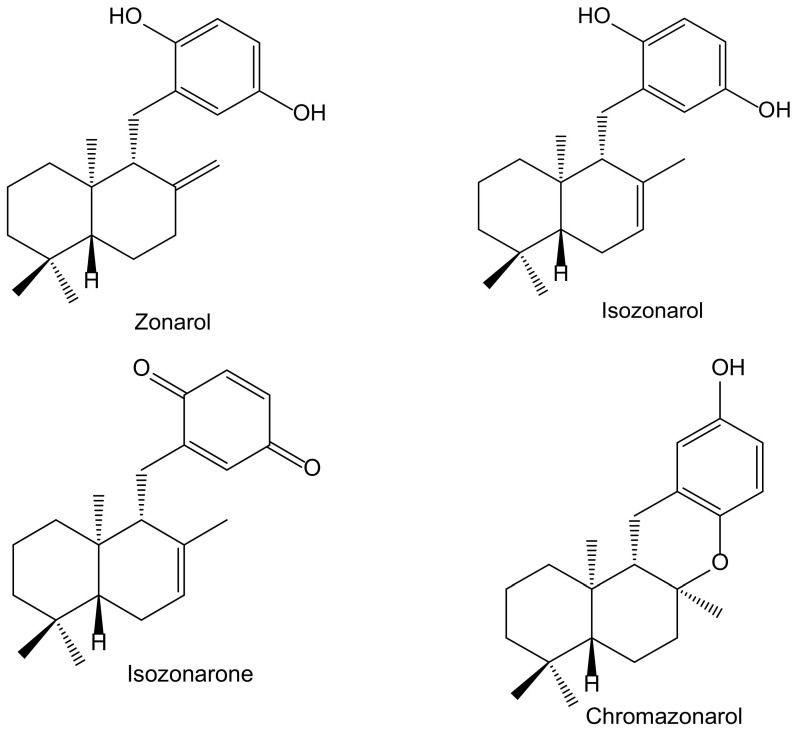
Chemical structures of hydroquinones and closely related derivatives isolated from *Dictyopteris undulata*.

In a screening for anti-oxidative substances, Kamagai et al. [14] found relevant activity of the methanol extract of the brown alga *Dictyopteris undulata* Holmes (Dictyotaceae) on the DPPH test. After a bioassay-guided fractionation, they isolated five sesquiterpenoids, three of them hydroquinone derivatives: isozonarol, isozonarone, and chromazonarol (Figure 8). These compounds were tested using the same protocol, and the authors observed that the DPPH^•^ radical scavenging activity was highest for isozonarol, with an EC_50_ of 71 μM, similar to that obtained for α-tocopherol used as a positive control. Isozonaroneand chromazonarol gave values of 145% and 121% of inhibition, respectively. In this case, it is clear that hydroquinone is the active center with efficient scavenging properties against ROS [15,16]. This relevant effect vanishes when the *p*-hydroquinone structure disappears or is blocked with different radicals. It was observed that other isolated sesquiterpenoids had no activity because they did not include the hydroquinone center in their molecules. As a conclusion, the authors proposed specific studies for establishing the potential anti-inflammatory and neuroprotective effects due to the structural similarity with zonarol, the activity of which was previously reported [14]. The latter compound was not tested in the DPPH test, but it was assayed on the nuclear factor-erythroid 2-related factor 2 antioxidant-responsive element (Nrf2/ARE) pathway for its potential neuroprotective effects. Indeed, zonarol protected HT22 hippocampal neuronal cells against oxidative stress, through activationof the Nrf2/ARE pathway. This compound can be considered a pro-electrophilic drug because the hydroquinone group can be oxidized to a quinone. The authors concluded that zonarol could be considered as an effective neuroprotective agent against oxidative insults, and mitochondrial dysfunction, and could be lead drug for the treatmentof chronic neurodegenerative diseases associated with oxidative stress [17].

**Figure 9:**
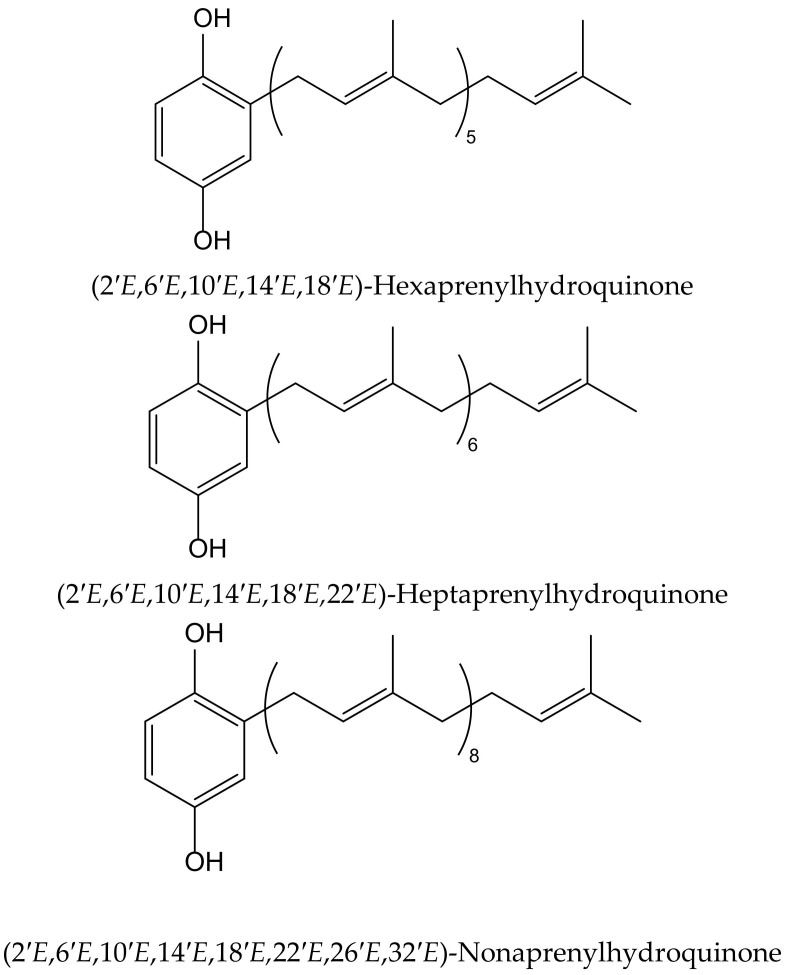
Chemical structures of hydroquinones isolated from *Sarcotragus muscarum*.

Wätjen et al. isolated three polyprenylhydroquinones from the Mediterranean marine sponges *Sarcotragus muscarum* (Schmidt, 1864) and *Ircinia fasciculata* (Pallas, 1766), both belonging to the family of the Irciinidae. These compounds were identified as hexa-, hepta- and nonaprenyl-1,4-hydroquinones (Figure 9). They displayed strong antioxidative activity, similar tothat ofTrolox, in a TEAC cell-free system, by using ABTS^•+^. Hexaprenylhydroquinone showed cytotoxicity at a low micromolar range (EC_50_ 2.5 μM) in H4IIE rat hepatoma cells. All three hydroquinones inhibited the activity of the nuclear factor-kappa B in this cell line, with heptaprenylhydroquinone being the most active. Hexa- and hepta-prenylhydroquinones showed inhibitory activity against certain protein kinases involved in signal transduction pathways, such as the epidermal growth factor receptor (IC_50_ 3.1 and 2.4 μM, respectively). Heptaprenylhydroquinone also inhibited the activity of Src tyrosine kinase, vascular endothelial growth factor receptor 3, and insulin-like growth factor 1 receptor [18].

**Figure 10:**
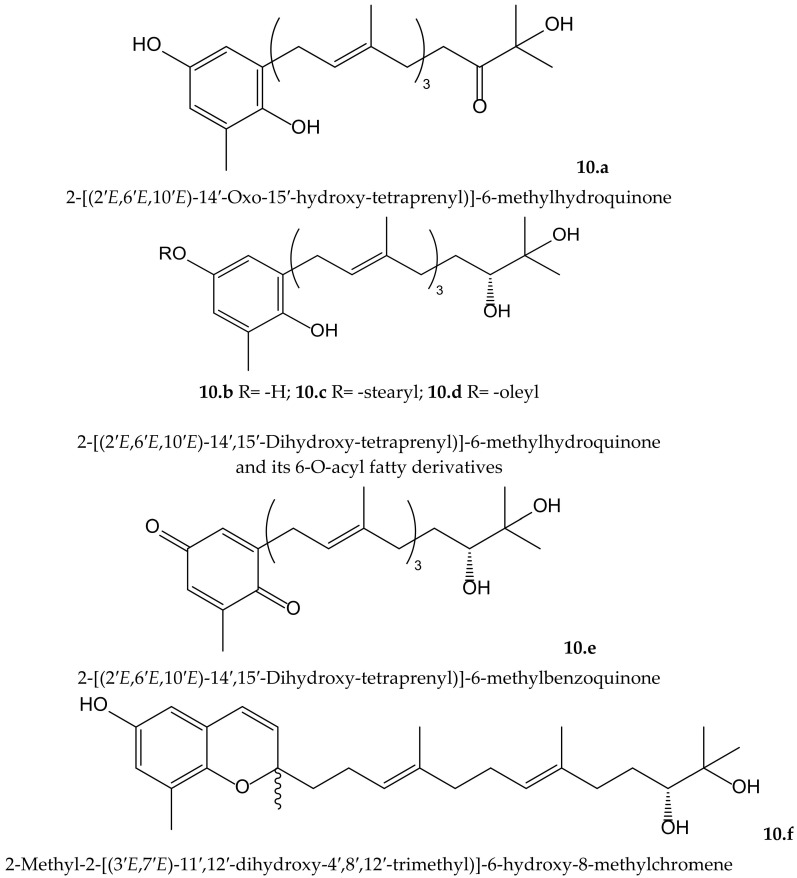
Chemical structures of hydroquinones and related compounds isolated from *Sargassum micracanthum*.

Mori et al. isolated four new plastoquinones (2-geranylgeranyl-6-methylbenzoquinones) (Figure 10) from the brown alga *Sargassum micracanthum* Kützing (Endlicher) (Sargassaceae), which showed radical scavenging effect on DPPH^•^ and inhibited NADPH-dependent lipid peroxidation in rat liver microsomes. Two of them (compounds **10.a** and **10.d**) showed IC_50_ values of 2.22 and 1.65 µMin lipid peroxidation, respectively, but they had only moderate reducing effect on DPPH^•^. It is of interest that the absence or presence of an unsaturated *cis* double bond in the fatty acid ester moiety of compounds **10.c** (stearyl) and **10.d** (oleyl), respectively, leads toa marked difference in the inhibitory activity. When authors tested the antiproliferative activity of these compounds, they observed that compounds **10.a** and **10.d** displayed interesting activity against colon 26-L5 cells with IC_50_ values of 3.52 and 2.43 µM, respectively, while compound **10.c** had only moderate activity (IC_50_ 25.12 µM) [19].

From the same species, Iwashima et al. isolated two plastoquinones and one new chromene derivative converted from plastoquinones (Figure 10). Two of the plastoquinones (**10.b** and **10.e**), and the chromene (**10.f**), exhibited significant antioxidant activities, with IC_50_ values of 0.25, 2.34, and 0.65 µM, respectively, on lipid peroxidation but a moderate radical reducing effect on DPPH^•^, with IC_50_ of 25.68 and 24.98 µM for compounds **10.b** and **10.f**, respectively. The strong antioxidant activity of these compounds might be related to the hydroquinone and phenol moiety, respectively, after their transformation in a stable phenoxyl radical [20].

**Figure 11:**
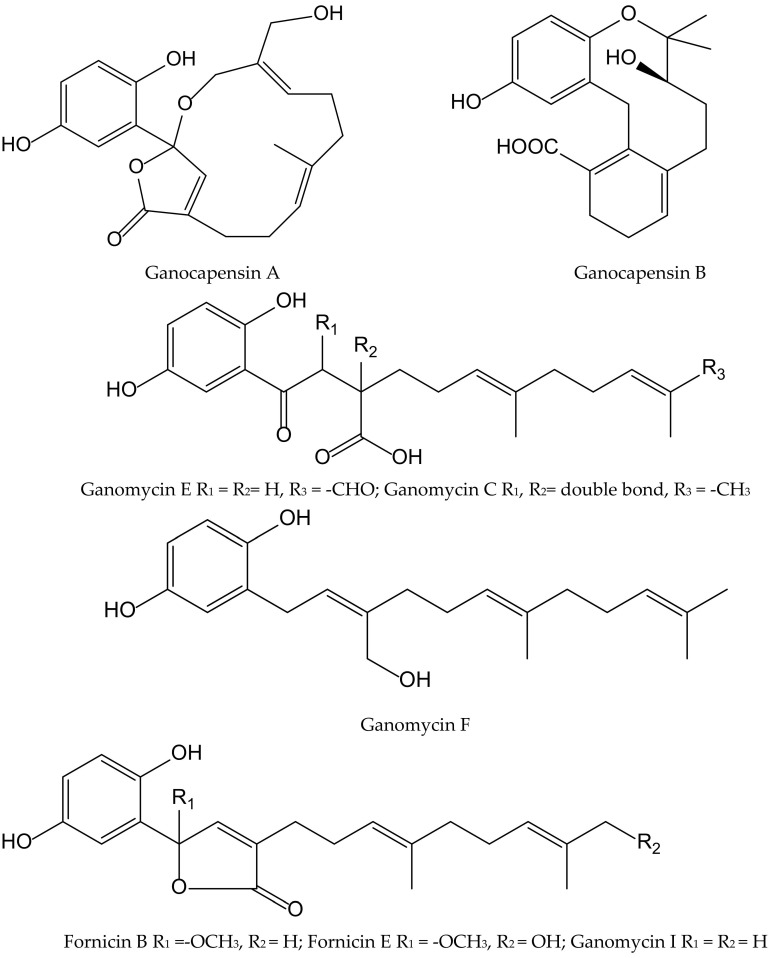
Chemical structures of hydroquinones isolated from *Ganoderma capense*.

The fungi of the Genus *Ganoderma* (Ganodermataceae) have been widely used as traditional Asian medicines for centuries and are gaining attention in Western countries. Phytochemical studies on this genus led to the isolation of hundreds of compounds, including over 380 terpenoids, e.g., ganoderic acids, lucidenic acids, and some meroterpenoids, which showed significant pharmacological activities such as antioxidant, immunomodulatory, and antitumoral properties. From the fruiting bodies of *Ganoderma capense* (Lloyd) Teng, eight hydorquinone meroterpenoids were isolated and identified as ganocapensins A and B; fornicin B and E; and ganomycin C, E, F and I (Figure 11).

All of them showed significant antioxidant effects in the DPPH^•^ radical scavenging assay, with IC_50_ values of 16–22 μM. The authors suggested that thein vitro antioxidant property of these aromatic meroterpenoids could be of interest for further research and could provide a theoretical basis to the application of compounds isolated from *Ganoderma* as anti-aging drugs [21].

### 2.3. Other Alkyl-Hydroquinones

**Figure 12:**
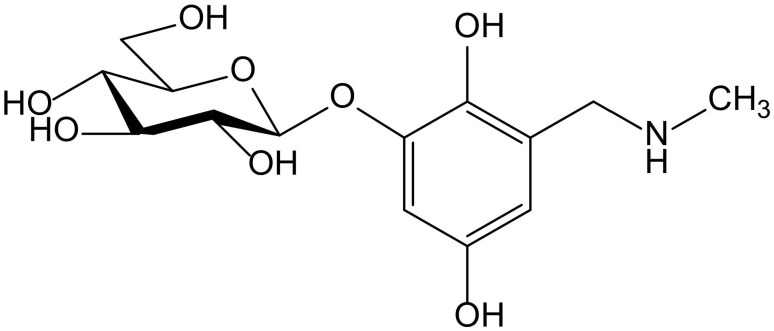
Chemical structure of zinolol isolated from *Anagallis monelli*.

Ammar et al. isolated a new hydroquinone glucoside from the aerial parts of *Anagallis monelli* L. (Primulaceae). The compound was identified as 2-(*O*-β-D-glucopyranosyl)-6-methylaminomethyl)hydroxy-hydroquinone and called zinolol (Figure 12). This compound was tested for its antioxidant activity on the ABTS and DPPH tests. Zinolol had its maximal inhibition at a concentration of 3 mM (98%) on the ABTS test after 5 min of incubation, although it also acted at low concentrations (0.09–1.5 mM) with inhibition percentages of 70% to 98% after 5 min of incubation, and these values were maintained. The reference compound Trolox produced a similar inhibition (98%, at 4 mM after 5 min of incubation). In the case of the DPPH^•^ scavenging capacity test, the antioxidant activity of zinolol was also 98% after 30 min of incubation, similar to Trolox (99% inhibition) in similar conditions (3 mM, after 30 min of incubation). The inhibitory effect of zinolol was concentration-dependent; its antioxidant effect increased from 26% (0.09 mM) to 97% (3 mM). This effect was due to the oxidation of hydroquinone skeleton and subsequent formation of the stable form of quinone. As a conclusion, the authors considered that zinolol is an effective antioxidant agent that could be useful for prevention of pathologies in which the free radicals and their oxidative damage are implicated, such as genotoxicity and cancer [22].

**Figure 13:**
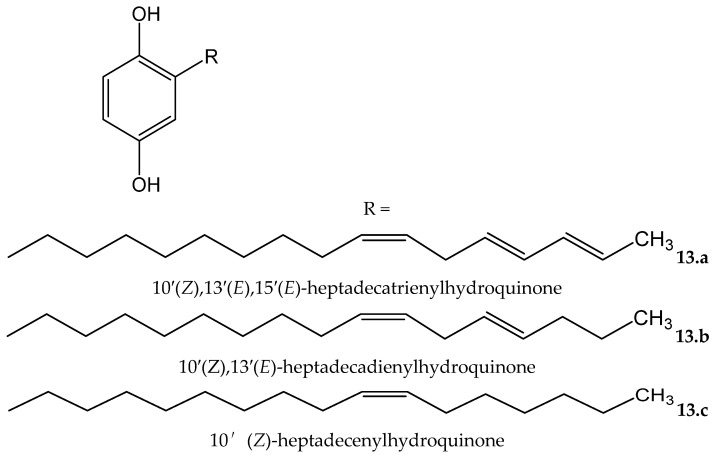
Chemical structures of hydroquinones isolated from *Rhus succedanea*.

*Rhus succedanea* L. (Anacardiaceae) is another source of hydroquinones. From the lacquer obtained of this plant, Wu et al. [23] isolated three compounds, two of them being new natural compounds, which were identified as: 10′(*Z*),13′(*E*),15′(*E*)-heptadecatrienylhydroquinone (**13.a**) and 10′(*Z*),13′(*E*)-heptadecadienylhydroquinone (**13.b**), as well as the known compound 10′(*Z*)-heptadecenylhydroquinone (**13.c**, Figure 13). All of them were tested in an iron/ascorbate system for establishing the capacity to inhibit peroxide formation in linoleic acid (used as a substrate). The antioxidative effectiveness was established for the three compounds, showing the two new compounds **13.a** and **13.b**, at 11.7 and 11.6 µM, respectively, near-complete inhibitory effects similar to the positive control, BHT, at 18.1 µM. Compound **13.c**, at 11,6 µM, reached 60% of the effect of BTH.

## 3. Interactions with Enzymes, Transcription Factors, and Other Proteins

Melanin is a natural skin pigment responsible for absorption and skin protection from harmful UV radiation. Tyrosinase plays an important role in melanin synthesis and is the main enzyme involved in the melanogenesis process in mammals and the enzymatic browning of fruits, vegetables, and fungi. Therefore, the search for new and potent tyrosinase inhibitors, including those of plant-based natural products, is of interest for application in medicine for the treatment of hyperpigmentation diseases, in cosmetics such as skin-lightening agents, and in the food industry. Several natural or synthetic compounds are used as active constituents of preparations to treat hyperpigmentation but are not completely adequate in terms of effectiveness and safety. In this context, one of the main depigmenting agents like hydroquinone is only available under prescription and banned in cosmetic use. Only a few compounds are currently used for clinical purposes as skin-whitening agents. Arbutin, characterized by its wide distribution in the Ericaceae and Caprifoliaceae families, exhibits anti-melanogenic activity in vitro and in vivo and can be useful in hyperpigmentation therapy [24]. The aqueous extract of *Arbutus andrachne* L. (Ericaceae) is used topically as skin lightener in some areas of Jordan. The methanol extract of the stems of this species, containing a high concentration of arbutin, exhibited 97% inhibitory activity against tyrosinase-catalysed oxidation of L-tyrosine [25].

**Figure 14:**
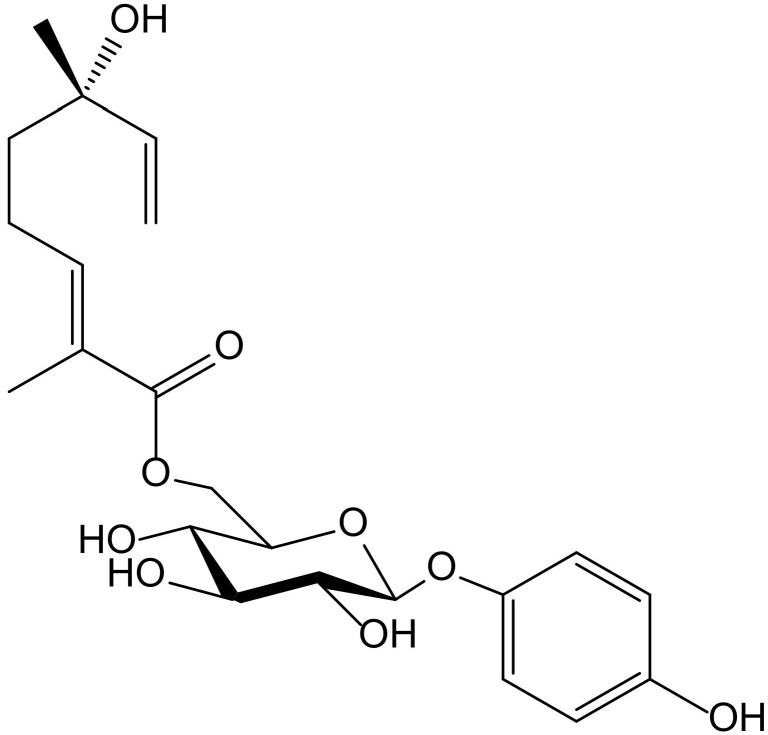
Chemical structure of phlebotrichin from *Viburnum erosum*.

Six new acyl-glucosides of hydroquinone containing different unsaturated linear monoterpenoid esters at the C-6″ of the sugar moiety, together with the known analogue phlebotrichin (Figure 14), were isolated from the leaves of *Viburnum erosum* Thunb (Caprifoliaceae). When evaluated for their tyrosinase inhibitory activities, these new derivatives did not exhibit any effect, probably due to the monoterpenoid chain that impedesbinding of tyrosinase enzyme to the compounds. Nevertheless, phlebotrichin still showed moderate effectiveness compared with the positive control arbutin [26].

Marine-derived and plant-pathogenic fungi are considered as a source of bioactive compounds that displayed a variety of pharmacological activities. Endophytic fungi inhabit plant tissues and are significant components of plant micro-ecosystems, altering the quality of the crude drugs through a specific fungus–host interaction since they can affect their host plants by improving their growth, enhancing their robustness, strengthening their tolerance to stress, and stimulating production of secondary metabolites.

**Figure 15:**
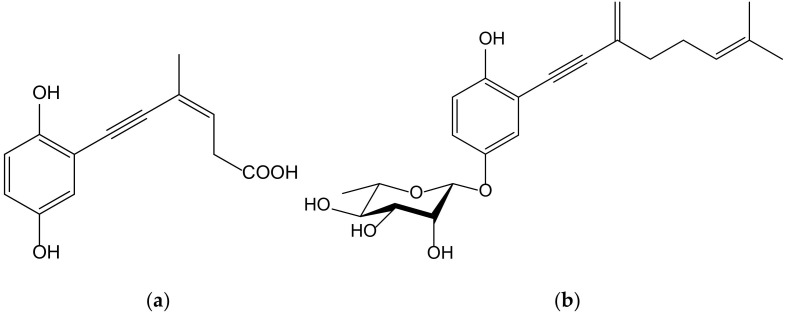
Chemical structure of pestalotioquinol C (**a**) and pestalotioquinoside C (**b**) isolated from a fermentation broth of *Pestalotiopsis neglecta,* a fungus endophytic to *Coelarthrum* sp.

Seven new terpenoid hydroquinones produced from the endophytic fungus *Pestalotiopsis neglecta* (Thüm.) Steyaert (Xylariaceae), strain SCSIO41403, were isolated from a *Coelarthrum* sp. red alga, collected at the sea coast of southern China. These compounds, bearing a vinyl alkyne, were identified as four ene-yne-hydroquinones, called pestalotioquinols C–F, and three glycosylated derivatives, the pestalotioquinosides A–C. The structure of pestalotioquinol C, which was quantitatively preponderant amongthe metabolites, was determined as 6(*Z*)-(2,5-dihydroxyphenyl)-4-methylhex-3-en-5-ynoic acid (Figure 15).

It has been reported that liver X receptor (LXR) α acts as oxysterol sensor; its agonists represent a feasible approach to control cholesterol efflux, and they are regulators of the reverse cholesterol transport pathway, preventing pathological conditions driven by lipid metabolism dysfunction, such as vascular and metabolic diseases, neurological degeneration, or cancer. The affinity of the abovementioned ene-yne-hydroquinones to the LXRα was analyzed in vitroand in silico, and the results demonstrated that pestalotioquinoside C (Figure 15) has LXRα modulatory properties. Through binding with the LXRα, this glycosylated hydroquinone (50 µM) stimulated the transcriptional activity driven by the receptor [27], which resulted in the upregulation of the gene *Abca1*, a key agent for the transmembrane transport of cholesterol and fatty acid derivatives, by interacting with Apo E and Apo A1 lipoproteins.

**Figure 16:**
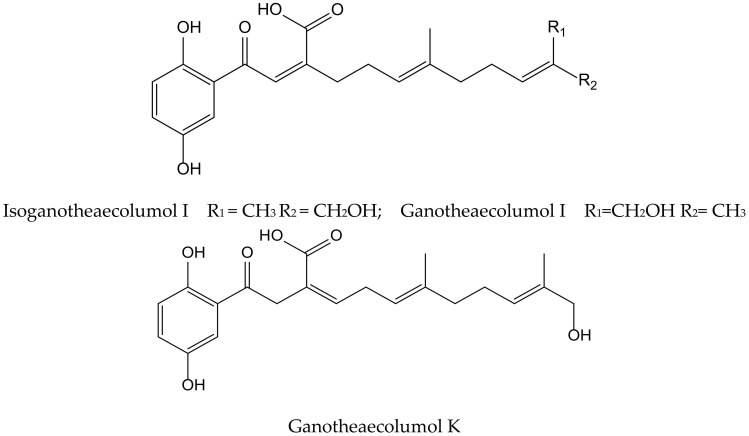
Chemical structures of hydroquinones isolated from *Ganoderma theaecolum*.

Luo et al. isolated seven new meroterpenoids containing a hydroquinone moiety, called ganotheaecolumols E–K, together with nine known compounds from the fruiting bodies of *Ganoderma theaecolum*, which is used as a substitute for *G. lingzhi* in the treatment of chronic diseases. The new derivatives, ganotheaecolumol I and K, and the known one, isoganotheaecolumol I (Figure 16), revealed inhibitory effects against cyclooxygenase-2 (IC_50_ values between 2.61 to 4.84 µM) and tyrosine-protein kinase 3 (IC_50_ values between 3.63 to 15.64 µM) [28].

**Figure 17:**
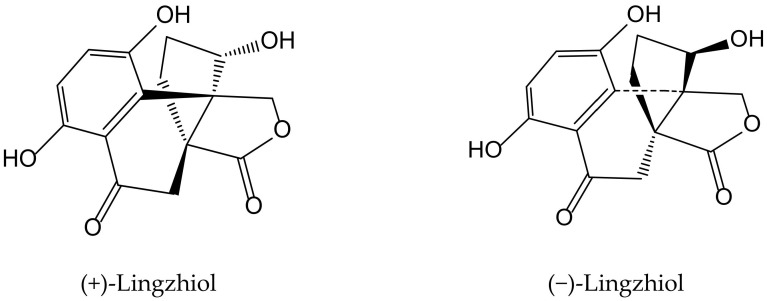
Chemical structures of hydroquinones isolated from *Ganoderma lucidum*.

Previously, Yan et al. [29] isolated a pair of meroterpenoid enantiomers from *Ganoderma lucidum* (Curtis) P. Karst., which were identified as (+)-lingzhiol and (−)-lingzhiol (Figure 17). Their pharmacological evaluation showed that both compounds at 10 and 30 µM significantly inhibited, in a concentration-dependent manner, ROS, collagen IV, fibronectin, and interleukin (IL)-6 overproduction in high-glucose treated mesangial cells. Under such diabetic-like conditions, both principles inhibited at 10 μM the transcript levels of collagen IV, fibronectin, and IL-6 but induced Nrf2, which is a key regulator of the cellular defense against electrophilic and oxidative species stress. (−)-Lingzhiol and, to a lesser extent, (+)-lingzhiol inhibited transforming growth factor-β_1_-mediated Smad3 phosphorylation but not that of Smad2, in a concentration-dependent manner in rat renal proximal tubular cells NRK-52E. All these findings suggest that both derivatives could have protective effects in kidney diseases. 

Several natural hydroquinones have been reported to be neuroprotective, suggesting that the ability of conversion of a *p*-dihydroxybenzene to a benzoquinone by autooxidation is essential for that activity because such compounds behave like pro-electrophilic compounds. They can activate Nrf2, which induces ARE-dependent expression of detoxifying and antioxidant defense proteins, such as NADPH quinone oxidoreductase 1 (NQO1), glutathione *S*-transferase, and heme oxygenase-1 (HO-1), maintaining the redox homeostasis of the cell.

**Figure 18:**
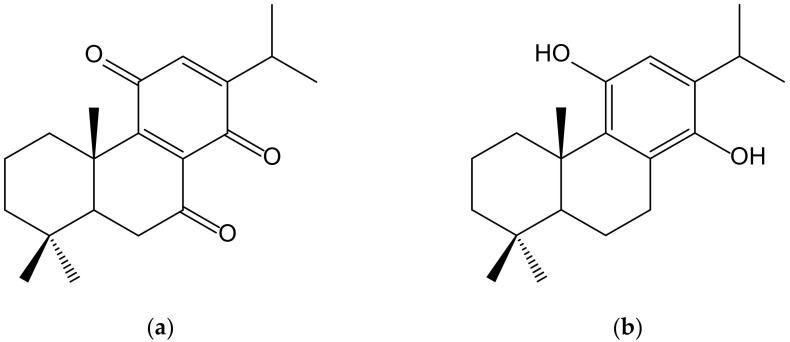
Chemical structures of cryptoquinone (**a**), isolated from *Cryptomeria japonica*, and of the synthetic hydroquinone, 11,14-dihydroxy-8,11,13-abietatriene (**b**).

Sasaki et al. [30] described the neuroprotective effects of a novel abietane-type diterpene *p*-hydroquinone (11,14-dihydroxy-8,11,13-abietatriene) (Figure 18) derived from cryptoquinone (7,11,14-trioxoabieta-8,12-diene) isolated from the bark of *Cryptomeria japonica* (Thunb. ex L.f.) D. Don (Cupressaceae). It activated, at 5 µM, the Nrf2/ARE system, inducing HO-1 and NQO1 expression as well as increasing glutathione in the neuronal HT22 cell line from mouse hippocampus, thus protecting neuronal cells from oxidative stress. Cryptoquinone exhibited cytotoxic activity against mouse lymphoid neoplasm cells P388 with an IC_50_ of 0.26 μM and modest antifungal activity against *Pyricularia orizae* and *Alternaria alternata* [31].

**Figure 19:**
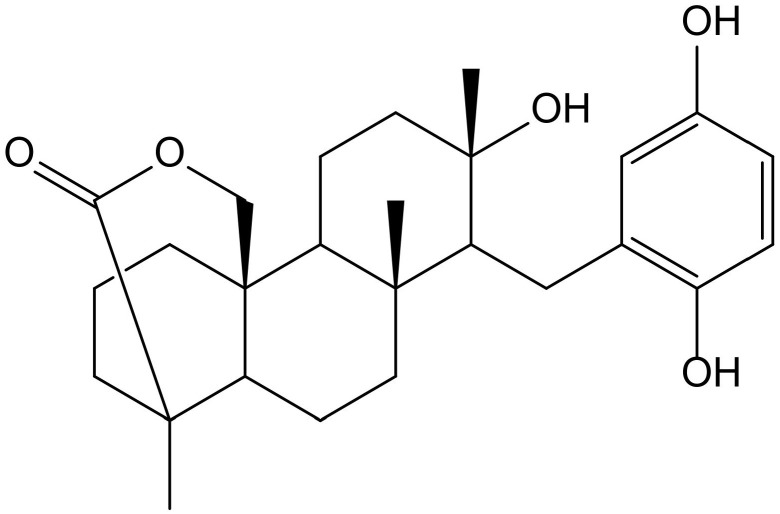
Chemical structure of strongylophorine-8, isolated from *Petrosia corticata*.

Previously, Sasaki et al. [32] demonstrated that strongylophorine-8 (Figure 19), a hydroquinone triterpene pro-electrophilic compound from the marine sponge *Petrosia (Strongylophora) corticata* (Wilson, 1925) (Petrosiidae), protected neuronal cells from oxidative stress by activating the Nrf2/ARE pathway, inducing phase 2 enzymes and increasing glutathione. However, its protective effects (4.1 µM) were very close to cytotoxic effects (14.24 µM), suggesting that it would not be adequate for further in vivo applications.

**Figure 20:**
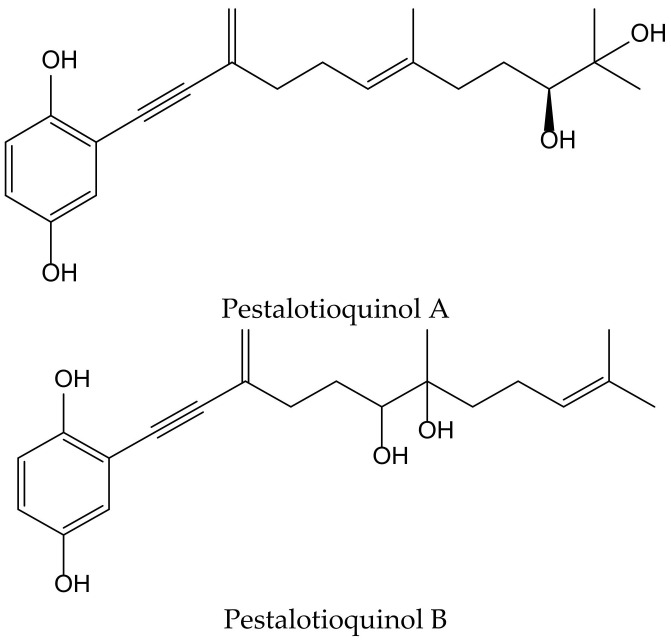
Chemical structures of hydroquinones isolated from *Pestalotiopsis microspora*.

Kanno et al. isolated the pestalotioquinols A and B (Figure 20) from a fungal culture broth of *Pestalotiopsis microspora* (Speg.) G.C. Zhao and N. Li. Pretreatment with 1–3 µM of pestalotioquinols A and B prevented the death of nerve growth factor-differentiated neuronal PC12 cells provoked by peroxynitrite (ONOO^−^), and the protective activity subsisted even after removing the compounds, showing neuroprotective effects [33].

**Figure 21:**
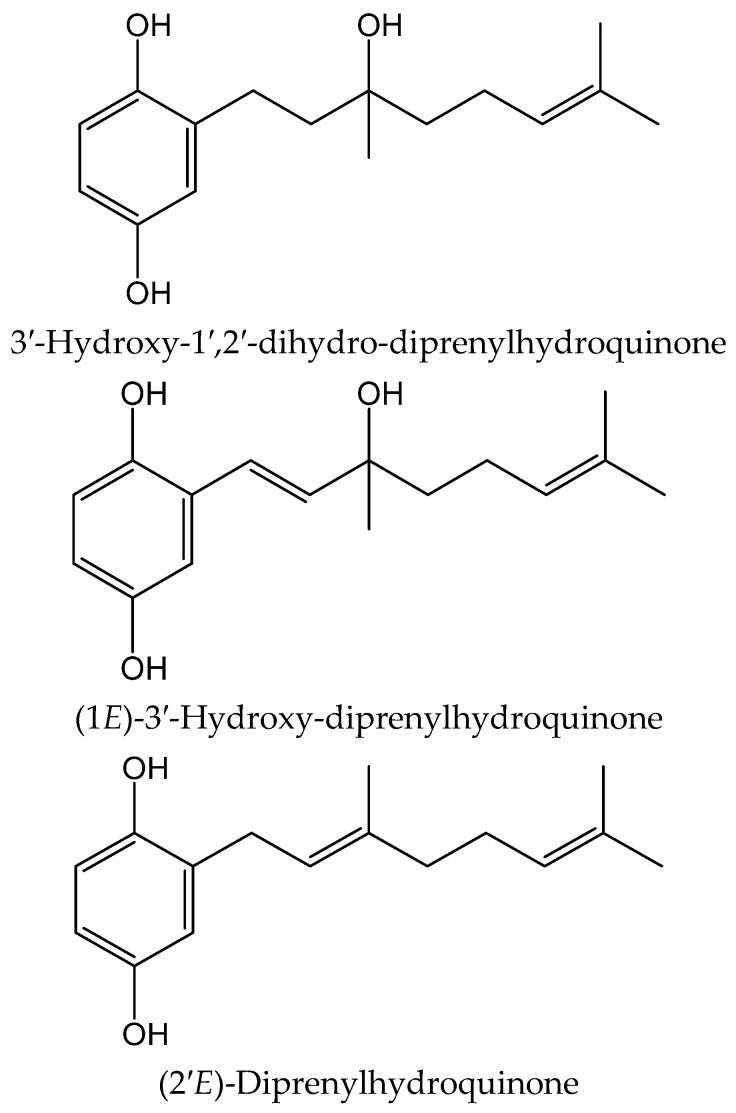
Chemical structures of hydroquinones isolated from *Amaroucium multiplicatum*.

From the colonial tunicate *Amaroucium multiplicatum* (Sluiter 1909, Polyclinidae) collected in central to southern Japan seashore, Sato et al. [34] isolated geranylhydroquinone and two new closely related hydroxyl derivatives. The novel compounds were identified as 3′-hydroxy-1′,2′-dihydro-diprenylhydroquinone, (1*E*)-3′-hydroxy-diprenylhydroquinone, and (2′*E*)-diprenylhydroquinone (Figure 21). These compounds were tested for their activity on soybean 15-lipoxygenase (15-LOX) and on lipid peroxide formation induced in rat liver microsomes by ferrous sulphate/cysteine. The 6-monoenyl derivative showed an inhibitory effect (IC_50_ 11.35 µM) in the latter one. Both new hydroquinones exhibited IC_50_ values of 1.14–3.78 µM in the former system, while only geranylhydroquinone produced IC_50_ values within the range 0.3–1.5 µM in both models. All three hydroquinones were more potent antioxidants than α-tocopherol acetate (IC_50_ > 21.15 µM).

**Figure 22:**
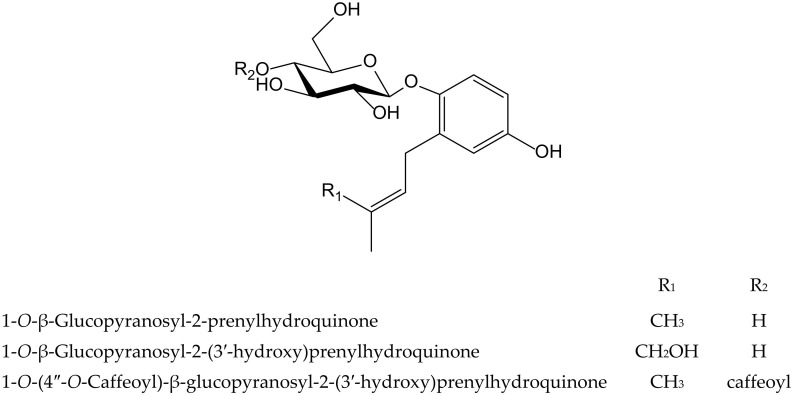
Chemical structures of hydroquinones isolated from *Phagnalon rupestre*.

Three new hydroquinone glucosides isolated from *Phagnalon rupestre* (L.) DC. (Compositae) were evaluated for their inhibitory effects on the nitration and oxidation of biomolecules induced by ONOO^−^. These compounds, 1-*O*-β-glucopyranosyl-2-prenylhydroquinone, 1-*O*-β-glucopyranosyl-2-(3′-hydroxy)-prenylhydroquinone, and 1-*O*-(4″-*O*-caffeoyl)-β-glucopyranosyl-2-prenylhydroquinone (Figure 22), inhibited the nitration of tyrosine by 79%, 49%, and 30%, respectively, at 100 µM, and the first one exhibited the highest potency (IC_50_ 40 µM). The benzene ring was susceptible to nitration, thus preventing that of tyrosine, but the presence of the hydroxymethyl group at the end of isoprenyl chain in the second compound, and a caffeoyl moiety in the third one, were unfavorable for such mechanism. However, all three compounds protected against the oxidation of dihydrorhodamine at 25 µM mediated by ONOO^−^, with caffeoyl derivative being the most active (IC_50_ 9.1 µM), as expected due to the presence of an *ortho*-dihydroxyl group [35]. The isoprenyl hydroquinone glucoside was further studied for its inhibitory activity against the protein carbonylationon caused by phorbol ester (12-*O*-tetradecanoylphorbol-13-acetate, TPA)-induced leukocyte oxidative burst, and on two cell-free models such as bovine serum albumin carbonylation caused by hypochlorite (OCl^−^) or ONOO^−^. In the latter one, the behavior of the compound on protein carbonylation was in line with its effect on tyrosine nitration described above. As expected, it was active in the presence of reactive nitrogen species and exhibited an IC_50_ value of 86.3 µM when the oxidation of protein was driven by ONOO^−^, but it showed no activity when the reaction was caused by OCl^−^. In the case of the respiratory burst of leukocytes stimulated with TPA, isoprenylhydroquinone glucoside was barely active, although it almost inhibited the effect at the high concentration of 100 µM [36].

## 4. Structure/Activity Relationship

To study the possible relationship between the chemical structure and the antioxidant activity, it is feasible to consider essentially the models included in Section 2 since, even with their deficiencies from a biological point of view, the methods applied are quite homogeneous and the experimental conditions controlled.For such a purpose, according to the reviewed literature, three determinations are definitely the most important: scavenging of DPPH^•^, scavenging of ABTS^•+^, and inhibition of lipid peroxidation. In the first case, the IC_50_ values of the different compounds ranged from 15 µM to 75 µM, with the most potent being simple hydroquinone and the hydroquinone meroterpenoids from *Ganoderma capense*, which are indeed quite different from each other. Regarding the inactivation of ABTS^•+^, only a few compounds have been described, although their potency (0.3–5.0 µM) is higher than that reported for the products assayed in the DPPH test. In both free radical scavenging systems, the lowest potency corresponds to the methylamine, zinolol. In the field of lipid peroxidation, numerous compounds with potency between 2.0 and 20.0 µM have been described, possessing in the vast majority of cases an alkyl, mostly prenyl, chain. A far from exhaustive comparison with some plant phenolics compounds [37] indicates that the reported hydroquinones have potencies in the range of well-known flavonols or catechins. According to the available data, it has not been possible to find additional criteria that could support a firm structural relationship with pharmacological potency.

## 5. Conclusions

Even for the least passionate student of the chemistry of natural products, it is very relevant, even magnificent, to observe the contrast between the enormous phylogenetic dispersion of the origin of raw materials, and the repetition of structural motifs. This occurs in a very obvious way with the wide range of antioxidant poly-isoprenylbenzenes and poly-isoprenylquinones. Probably because hydroquinones have relatively little value as active principles in medicinal plants, most reports of their antioxidant activity lack a direct connection to potential therapeutic value. This is because many articles, otherwise very interesting from a chemical point of view, contain only a fairly brief account of the possible bioactivity of the compounds studied. However, some lines of pharmacological progress are easily perceivable in the literature analyzed. As an example, the neuroprotective activity of zonarol and analogs from *Dictyopteris*, based on the stimulation of the Nrf2/ARE pathway, deserves special mention. Moreover, the uniqueness of the chemistry of aromatic ene-ynes pestaloquinol analogs, together with their agonistic interaction with LXR α, proved to be of high value for a screening in the pharmacology of atherosclerosis. Finally, the dissection of indiscriminate antioxidant activity from selective effects on tyrosine nitration or protein carbonylation, as occurs in the principles of *Phagnalon*, presents interesting possibilities in the control of post-translational protein modification habitually appearing in chronic inflammatory diseases.

## Abbreviations

Acronyms, symbols and formulae.

ABAP	2,2′-azobis(2-amidinopropane)
ABTS	2,2′-azinobis(3-ethylbenzothiazoline-6-sulfonic acid)
BHT	butylated hydroxytoluene
DPPH	1,1-diphenyl-2-picrylhydrazyl
EC_50_	half maximal effective concentration
EDTA	ethylenediaminetetraacetic acid
FRAP	ferric reducing antioxidant power
HO-1	heme oxygenase-1
IC_50_	half maximal inhibitory concentration
IL	interleukin
15-LOX	15-lipoxygenase
LXRα	liver X receptor α
NQO1	NADPH quinone oxidoreductase 1
Nrf2/ARE	Nuclear factor-erythroid 2-related factor 2/antioxidant-responsive element
O^2•^^−^	superoxide anion
OCl^−^	hypochlorite anion
ONOO^−^	peroxynitrite anion
PHB	*p*-hydroxybenzoate
ROS	reactive oxygen species
SC_50_	scavenger concentration 50%
TBARS	thiobarbituric acid reacting species
TEAC	Trolox equivalent antioxidant capacity
Trolox	6-hydroxy-2,5,7,8-tetramethylchroman-2-carboxylic acid
